# Computer-based testing in higher education: a phenomenology investigation into undergraduate students’ perspectives through the technology acceptance model

**DOI:** 10.3389/fpsyg.2026.1602964

**Published:** 2026-01-22

**Authors:** Yusuf Feyisara Zakariya, Sarah Bader Alotaibi, Jawaher Saud Alrashood, Tahani Mohammed Alrosaa

**Affiliations:** 1 Department of Science Education, Ahmadu Bello University, Zaria, Nigeria; 2 Department of Mathematical Sciences, University of Agder, Kristiansand, Norway; 3 Department of Teaching and Learning, College of Education and Human Development, Princess Nourah Bint Abdulrahman University, Riyadh, Saudi Arabia

**Keywords:** attitudes toward computer-based testing, behavioral intention, CBT anxiety, ChatGPT, students’ performance, technology acceptance model

## Abstract

**Background:**

Computer-based testing (CBT) is widely adopted in Nigerian higher institutions, with even some national pre-university examinations now conducted through CBT. However, an in-depth investigation into the perceptions of users, especially students, is lacking.

**Objective:**

This study, employing a phenomenological approach within the qualitative research paradigm, examines university students’ perceptions of CBT through the lens of the Technology Acceptance Model.

**Methods:**

Using a self-developed interview protocol, we generated data through semi-structured individual interviews of undergraduate students purposively selected for this study. We analyzed the generated data using thematic analysis aided by ChatGPT-4o.

**Results:**

The results of this study show that university students viewed CBT as useful for fast grading and reduced administrative errors, though technical failures and rigid multiple-choice formats limited its perceived value. Ease of use was generally positive, especially among digitally skilled students, but server crashes and poor facilities undermined usability. Attitudes toward CBT were mixed, shaped by efficiency, fairness concerns, and exam-related stress. Behavioral intention to continue using CBT remained conditional: students were willing to adopt it if reliability, system support, and assessment flexibility improved. These results are consistent with TAM’s emphasis on usefulness, ease of use, and favorable attitudes in shaping technology acceptance.

**Conclusion:**

The findings have two key implications. First, universities should implement structured digital literacy training to ensure all students can navigate CBT effectively, reducing anxiety and improving confidence. Second, assessment policies should be refined to enhance fairness by incorporating diverse question formats and addressing inconsistencies in difficulty levels. We argue that addressing these concerns will improve student trust and engagement with CBT. Finally, we proposed a framework for AI-assisted qualitative data analysis as a contribution to the literature, among others.

## 1 Introduction

Assessment in higher education is a crucial pedagogical component designed to evaluate students’ understanding, competencies, and overall learning progression. Research (e.g., Black and Wiliam, 2009; Harlen and James, 1997; Schellekens et al., 2021) show that assessments serve both formative and summative purposes, which enable teachers to provide feedback that enhances learning while measuring academic achievements. Formative assessments, such as quizzes and assignments, support students in identifying their strengths and areas requiring improvement. On the other hand, summative assessments, including final exams, validate students’ knowledge and acquired skills (Schellekens et al., 2021). Moreover, assessments help institutions gage the effectiveness of curricula and teaching strategies to ensure alignment with educational standards and accreditation requirements (Biggs et al., 2022). Studies (e.g., Baas et al., 2015; de Vries et al., 2022; Zakariya et al., 2022) have shown that well-structured assessment systems enhance students’ metacognitive abilities, encouraging deep learning approaches rather than rote memorisation. Additionally, assessment outcomes influence key academic decisions such as progression, certification, and career preparedness. The advent of digital technology has further transformed assessment practices, enabling diverse approaches such as online quizzes, digital portfolios, and computer-based testing, which offer efficiency and scalability (Baartman and Quinlan, 2023; Sangwin, 2013). As higher education continues to evolve, integrating innovative assessment methods remains essential in preparing students for 21st-century challenges.

Building on the centrality of assessment in higher education, it is important to consider how assessment modalities have evolved in response to technological advancements. The modes of assessment have evolved from traditional paper-based examinations to more technologically driven formats like computer-based testing (CBT). CBT is an assessment method in which test items are delivered, completed, and scored using a computer or digital device rather than traditional paper-and-pencil formats. It uses specialized software to present questions, record responses, and automatically score or store results, making testing faster, more efficient, and easier to administer. Paper-based assessments have historically been the primary mode of evaluation, offering familiarity and minimal technological barriers. However, they have drawbacks such as logistical challenges, high costs of paper printing, and delays in result processing (Pengelley et al., 2025; Yeboah, 2023). On the other hand, CBT has gained traction due to its efficiency, automation, and potential to enhance academic integrity through randomized question banks and automated grading (Pengelley et al., 2025; Weirich et al., 2024). While CBT presents numerous advantages, such as instant feedback, reduced administrative burden, and flexibility in question formats, it also has limitations. Studies (e.g., Adubika and Agashi, 2021; Malec, 2020) indicate that students unfamiliar with digital platforms may experience anxiety, which could affect their performance. Additionally, network connectivity issues and cybersecurity threats pose risks to CBT implementation, particularly in resource-constrained environments (Abba and Abubakar, 2020; Perry et al., 2022). In contrast, paper-based assessments allow for subjective and qualitative evaluations, such as essay-based responses, which remain challenging for CBT to grade effectively (Pengelley et al., 2025). Given these strengths and limitations, it becomes necessary to examine how CBT is increasingly shaping assessment practices globally.

The increasing popularity of CBT in higher education is driven by its efficiency, adaptability, and ability to align with contemporary educational needs. Several empirical studies (e.g., Perry et al., 2022; Syahbrudin et al., 2025) have highlighted its affordances, including improved accessibility, enhanced security, and the ability to facilitate large-scale standardized testing. Particularly in institutions experiencing rapid student enrolment growth, CBT offers a scalable solution for managing assessments without compromising quality (Syahbrudin et al., 2025). Moreover, the COVID-19 pandemic accelerated the shift toward digital assessments, further normalizing CBT in higher education institutions. Studies (e.g., Pengelley et al., 2025; Yeboah, 2023) also indicate that students perceive CBT as more convenient due to its flexibility and quick result processing. A scoping review of studies by Morris and Rohs (2021) found that students who engage in digital assessments demonstrate higher levels of self-directed learning and digital literacy. Additionally, adaptive testing mechanisms within CBT platforms allow for a personalized learning experience which caters to individual student needs (Dai et al., 2022). These affordances of CBT have fuelled its adoption in higher institutions worldwide, in which, Nigeria is not an exception.

Despite the widespread adoption of CBT in many higher education institutions in Nigeria, research on its implementation and effectiveness remains limited. Most available studies (e.g., Azor and Edna, 2019; Shobayo et al., 2023; Usman and Olaleye, 2022) are survey-based using poorly validated instruments which offer surface-level insights without an in-depth exploration of pedagogical and technological implications of CBT. Beyond methodological limitations, a significant number of these studies (e.g., Shobayo et al., 2023; Usman and Olaleye, 2022) on CBT in Nigeria is either atheoretical or misapplies theoretical models that fail to capture the complexity of technology acceptance in education. For instance, some studies (e.g., Ukwueze and Uzoagba, 2021) adopt general ICT adoption frameworks instead of the Technology Acceptance Model (TAM), which specifically explains students’ and educators’ attitudes toward digital assessments (Davis and Granić, 2024). The TAM model posits that users’ adoption of a new technology is influenced by their attitude toward the technology which, in turn, is predicted by the perceived usefulness and perceived ease of use of the technology (Davis and Granić, 2024). In the context of CBT, this means students are more likely to embrace digital assessments if they find them user-friendly and beneficial to their learning outcomes. However, studies applying TAM in Nigeria’s higher education sector remain scarce, limiting insights into student engagement, technological readiness, and performance outcomes. Addressing these gaps requires more rigorous, theory-driven research to inform evidence-based policymaking and sustainable CBT implementation strategies.

Thus, the present study aims to explore undergraduate students’ experience of CBT through the lenses of TAM within the context of the qualitative research paradigm. This exploration covers the perceived usefulness, ease of use, attitudes to use, and behavioral intention toward CBT among university students in Nigeria. To achieve this aim, this study addresses the following research questions:

1. What is the perceived usefulness of CBT among university students?
2. What is the perceived ease of use of CBT among university students?
3. What are the attitudes of university students toward CBT?
4. What is the behavioral intention of university students to use CBT?

Addressing these research questions is crucial for understanding how university students perceive and engage with CBT in Nigeria. Examining perceived usefulness helps determine whether students believe CBT enhances academic performance, improves efficiency, saves time, and provides a better testing experience. Investigating perceived ease of use identifies usability challenges that may hinder adoption. Exploring students’ attitudes toward CBT provides insights into their preferences, concerns, and emotional responses to digital assessments. Lastly, analyzing behavioral intention to use CBT predicts future adoption trends. We argue that our attempt at investigating these key areas of students’ engagement with CBT with a theory-driven approach, particularly through the TAM, will ensure effective policy formulation and sustainable CBT implementation in Nigerian higher education. Furthermore, it is important to remark that TAM was selected for this study because of its constructs, perceived usefulness, perceived ease of use, attitude toward use, and behavioral intention, which directly reflect the cognitive processes that underpin students’ engagement with CBT. Unlike TAM2, TAM3, and the unified theory of acceptance and use of technology (Davis and Granić, 2024), which incorporate broader social, organizational, and facilitating conditions, TAM offers a more parsimonious and education-focused analytical lens. This simplicity enhances interpretive clarity, allowing the qualitative data to illuminate how students’ lived experience with CBT naturally map onto TAM constructs, thereby generating coherent, context-grounded explanations of technology acceptance in assessment environments.

## 2 Materials and methods

### 2.1 Research design

**Figure 1:**
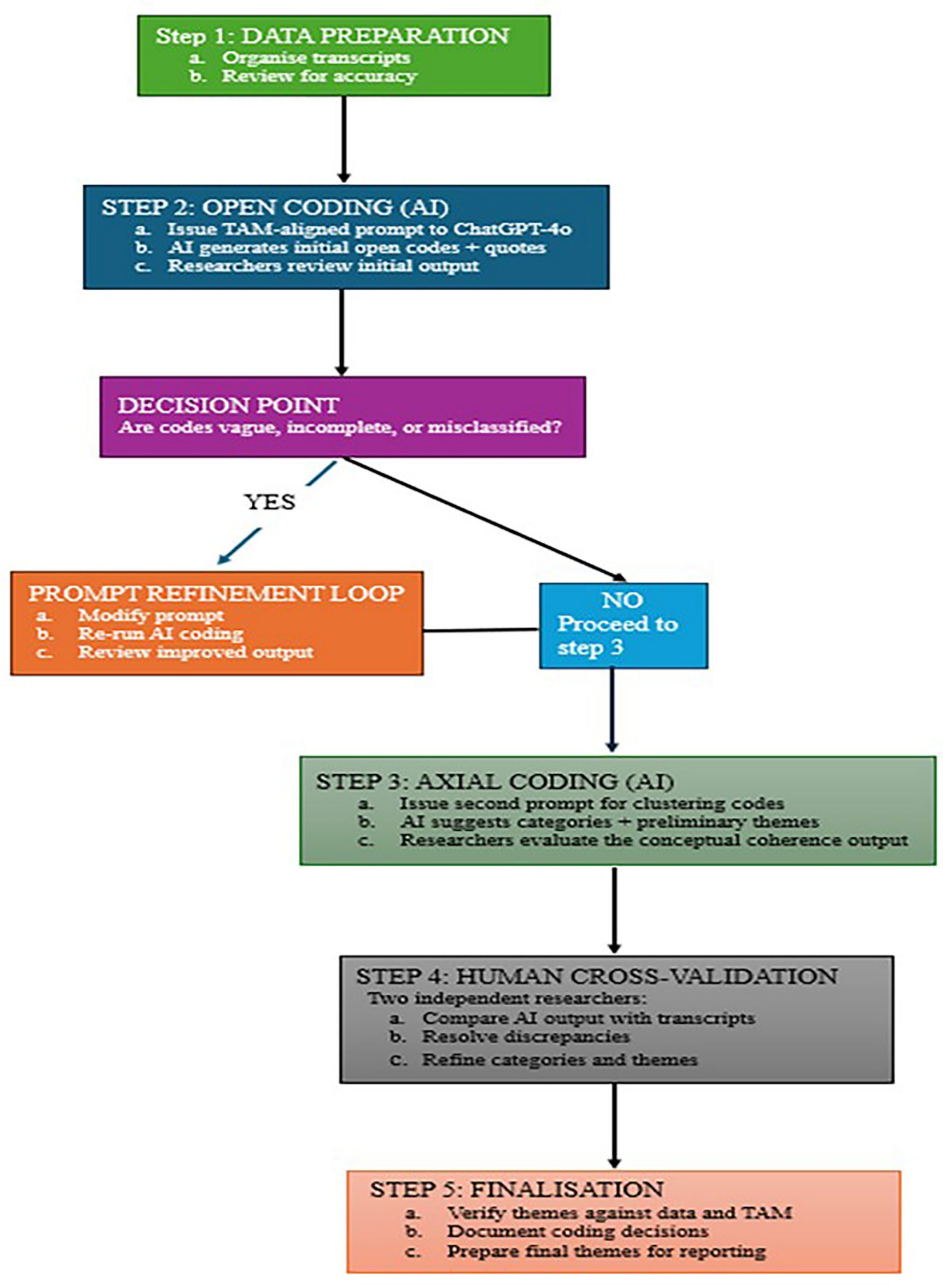
AI-assisted qualitative data analysis framework.

This study employed a phenomenological approach within the qualitative research paradigm (Creswell and Creswell, 2017) to explore undergraduate students’ experiences with CBT. According to Creswell and Creswell (2017), phenomenology is used in qualitative research to examine and describe individuals’ lived experiences with a particular phenomenon. It emphasizes capturing the essence of these experiences from participants’ perspectives without imposing preconceived assumptions. Thus, the phenomenological approach is appropriate for this study, as we seek to understand university students’ perceptions of the usefulness, ease of use, attitudes toward, and behavioral intentions regarding CBT (Figure 1).

### 2.2 Participants

A purposive sampling technique was used to select nine undergraduate students (six males and three females) who had prior experience with CBT at the largest university in Sub-Saharan Africa. This non-probability sampling method was appropriate as the study aimed to explore students’ lived experiences with CBT through the lens of TAM. A sample of nine participants was deemed sufficient for this qualitative study because it allows for in-depth exploration of experiences, ensuring rich, detailed data. In qualitative research, the focus is on depth rather than breadth, and smaller (8–12 participants), purposively selected samples can achieve data saturation, providing meaningful insights while maintaining manageability and enabling thorough, context-sensitive analysis (Braun et al., 2019). The sample included students from diverse academic disciplines and levels, which ensures a broad representation of the perspectives we sought to explore. Some participants are in their 100-level of studies while others are in their final year. Table 1 presents pseudonyms, gender, departments, and students’ levels of study.

**Table 1:** Undergraduate students’ pseudonyms and other biodata.

SN	Name	Gender	Department	Level
1	Aisha Umar	Female	English and Literary Studies	400 level
2	Joy Isa	Male	Political Science and International Studies	Not specified
3	Musa Kareem	Male	Computer Science Education	400 level
4	Mark Douglas	Male	Mass Communication	100 level
5	Patience Vijay	Female	Physical and Health Education	Not specified
6	Maryam Abubakar	Female	Computer Science	Not specified
7	Bosun Ahmad	Male	Electrical Engineering	100 level
8	Kola Tesslyn	Male	Agricultural Education	400 level
9	Akeem Salawu	Male	Science Education	400 level

Table 1 presents the demographic details of nine undergraduate students from the same higher institution, who participated in this study. The table shows that four students are in their final year (400 level) in the departments of English and Literary Studies, Computer Science Education, Agricultural Education, and Science Education. Two students are at the 100 level, studying Mass Communication and Electrical Engineering. Three students did not specify their academic level. The representation of students from various faculties and academic departments provides a broad perspective on CBT. We argue that this diversity in academic background and experience with CBT enriched the study’s exploration of perceived usefulness, ease of use, attitudes toward, and behavioral intentions of undergraduate students regarding CBT adoption in higher education.

### 2.3 Data collection

The data collection process involved conducting individual semi-structured interviews with the undergraduate students, guided by a self-developed interview protocol. The protocol was designed based on the TAM with some insights from the literature, while adhering to best practices in qualitative research (Braun and Clarke, 2013). The interview guide consisted of open-ended questions categorized into four key areas: perceived usefulness, perceived ease of use, attitudes toward CBT, and behavioral intention to use CBT. Each interview lasted approximately 17–22 min and was conducted in a quiet setting to ensure clarity and participant comfort. The interviews were audio-recorded with participants’ consent and later transcribed verbatim for thematic analysis (Braun et al., 2019). All participants provided written consent before participation, agreeing to the recording of their responses and the use of the transcribed data for research purposes. To maintain neutrality and data reliability, the interviews were conducted by trained research assistants who were students at the university but were not part of the study sample. Their role was to facilitate open discussions while ensuring that students’ perspectives on CBT were accurately captured. This rigorous approach ensured that the data collected reflected diverse experiences and attitudes toward the implementation of CBT in higher education.

### 2.4 Data analysis

The data analysis for this study followed a comprehensive, multi-step approach based on the guidelines set by Creswell and Creswell (2017). Initially, the data was organized and prepared for analysis by structuring the interview transcripts in a way that aligned responses with each specific interview question. This process involved rearranging the transcribed raw data from transcripts per student in nine Microsoft Word documents into transcripts across students’ responses to questions in each key area of the interview protocol using four Microsoft Word documents. The organized data was reviewed thoroughly to familiarize ourselves with the content. This stage of preparation was crucial for ensuring that all aspects of the students’ experiences with CBT were accurately captured. The next phase involved coding, where we identified recurring patterns and themes within the data. Instead of using traditional manual coding methods or standalone software such as NVivo, we utilized ChatGPT-4o for open and axial coding. To enhance methodological transparency and facilitate replicability, we provide additional details on the implementation of AI-assisted qualitative coding. For each research question, we developed structured prompts aligned with the relevant construct of the TAM, which guided ChatGPT-4o in generating the initial open codes. These prompts instructed the system to identify meaningful units of data, extract direct participant quotations, and produce discrete codes grounded in the transcripts. The AI-generated outputs were not adopted automatically; rather, an iterative approach was employed. The research team examined each set of open codes for accuracy, relevance, and conceptual alignment with the raw data. Where ambiguities or overly broad codes were observed, the prompt was refined and reissued to obtain more precise and differentiated coding.

Following open coding, a second set of prompts was used to carry out axial coding. These prompts directed the system to cluster the refined codes into coherent categories and propose potential themes consistent with the theoretical framework and objectives of the study. The resulting categories and themes were then subjected to a rigorous human review process. Two independent researchers cross-validated the AI-generated outputs against the verbatim transcripts, resolved discrepancies through discussion, and made further refinements where necessary. This multi-stage, researcher-supervised procedure ensured that the coding process remained systematic, transparent, and grounded in participants’ perspectives, while reducing the possibility of bias or misinterpretation arising from AI-generated suggestions. We provide further details on this AI-assisted qualitative analysis framework including associated prompts in Appendix A. This AI-assisted qualitative data analysis was performed separately for each group of students: those in the first year and those in their final year, while those who did not specify their levels were randomly classified into either of the two levels. This procedure allowed us to explore any differences in perceptions based on the academic level of the participants. Finally, a meta-analysis of the combined results was conducted, integrating the findings from both student groups to offer a coherent narrative for the study. The final themes that emerged from this cross-validation process are presented in the subsequent sections. These results provide valuable insights into undergraduate students’ attitudes toward, perceptions of, and intentions to use CBT in their academic assessments.

The decision to incorporate AI-assisted coding in this study was informed by the growing recognition of large language models as valuable analytical tools in qualitative research. AI systems such as ChatGPT-4o can efficiently identify preliminary patterns in large textual datasets and support researchers in generating initial codes in a structured and timely manner. In this study, the AI tool served as a complementary analytic resource rather than a replacement for human interpretation. Its role was limited to producing initial coding suggestions based on explicit, theory-driven prompts, while all interpretive decisions, refinements, and theme finalization were undertaken by the research team. The use of AI facilitated analytical efficiency and enhanced reflexivity by allowing researchers to compare their interpretations with machine-generated alternatives. Through careful supervision and multi-stage cross-validation, the approach balanced innovation with methodological rigor, ensuring that the analysis remained grounded in the participants’ lived experiences.

## 3 Results

### 3.1 Addressing the research question one on the perceived usefulness of CBT among university students

By perceived usefulness, we mean the degree to which students believe CBT enhances academic performance, improves efficiency, saves time, and provides a better testing experience. Through qualitative analysis using open and axial coding, three major themes emerged. These themes reflect students’ perspectives regarding the benefits and challenges of CBT concerning their academic performance, ease of use, and effectiveness. Direct quotes from the interviewees are used to support the findings, and interpretations are made in line with the TAM framework.

#### 3.1.1 Theme 1: Mixed user experience with CBT

The first major theme captures the varied experiences of students in using CBT. While some students found CBT convenient and easy to use, others encountered technical challenges that affected their overall perception. This theme is significant because user experience plays a vital role in determining technology acceptance, as proposed in TAM.

##### 3.1.1.1 Negative experiences due to technical challenges

Students reported negative experience with CBT due to technical failures such as poor network connectivity and system malfunctions. For instance, Aisha expressed her frustration:

*"My general experience with CBT has not been great. I feel they can do better with the network system because network issues disrupt the entire process. So, I believe they should improve it."*

This aligns with TAM’s assumption that if a system is unreliable or difficult to use, its perceived usefulness decreases (Davis and Granić, 2024). Technical disruptions not only hinder students’ ability to complete exams smoothly but also increase their test anxiety, making them less likely to prefer CBT over traditional paper-based tests.

##### 3.1.1.2 First-time anxiety vs. gradual adaptation

Another key aspect of students’ experience with CBT was the initial anxiety associated with their first encounter with the system. For example, Joy recalled her first experience:

*"My first experience with CBT was in 2023 when I took my JAMB exam. At first, I felt tense because it was my first time taking a CBT exam. However, as I proceeded, it became smooth and easy."*

This highlights how familiarity with technology plays a role in perceived usefulness. According to TAM, as students gain experience with the system, their perception of its usefulness improves. Joy’s experience shows that although she initially struggled with anxiety, she eventually found the system manageable and smooth, indicating a learning curve.

##### 3.1.1.3 Limited exposure to CBT in university settings

Some students pointed out that their exposure to CBT was limited, which impacted their ability to fully embrace it. Aisha stated:

*"At most, I use CBT once a semester. I only use it for general courses."*

Similarly, Maryam remarked:

*"Well, my experience with computer-based testing hasn't been extensive because I have used paper-based exams more frequently."*

This suggests that inconsistent exposure to CBT may affect students’ confidence and willingness to embrace the system fully. If CBT were integrated into more assessments, students might find it more useful and easier to navigate over time.

#### 3.1.2 Theme 2: Perceived limitations in academic assessment

The second major theme relates to how students perceive the effectiveness of CBT in assessing their knowledge. Many students questioned its ability to accurately measure academic performance, particularly because of its reliance on multiple-choice questions (MCQs).

##### 3.1.2.1 The multiple-choice format limits expression

Several students criticized CBT for limiting their ability to express themselves, which impacted their performance. Musa stated:

*"I don’t think CBT necessarily improves academic performance. This is because CBT often uses multiple-choice questions, whereas paper-based tests allow for more open-ended responses where I can express my ideas more freely."*

Similarly, Patience emphasized:

*"With traditional paper-based tests, I can write my answers in a way that even if my final answer is incorrect, I can still earn marks for relevant points. However, with CBT, it is just a straightforward answer. The examiner cannot tell whether I understood the concept or not."*

According to TAM, for students to perceive technology as useful, it must enhance their performance (Davis and Granić, 2024). However, if they believe that CBT restricts their ability to showcase knowledge, they may be less likely to view it as beneficial.

##### 3.1.2.2 Encouraging guesswork over understanding

Some students believed that CBT promoted guesswork rather than actual learning. Joy argued:

*"If I were a lecturer, I would prefer paper-based tests because they allow for better assessment of students. Many students rely on guesswork in CBT rather than actually knowing the material."*

Kola similarly stated:

*"Most CBT tests are objective (multiple-choice questions), which makes them easier. Since there is a 1/4 chance of selecting the correct answer, it simplifies the exam process for me."*

This suggests that students may perceive CBT as less effective in measuring true academic competence. If students do not see a direct link between CBT and academic improvement, its perceived usefulness diminishes in line with TAM.

#### 3.1.3 Theme 3: Efficiency vs. time constraints

This final theme focuses on the time management aspect of CBT and its efficiency for both students and lecturers. While CBT offers speed and automation, it also introduces challenges related to rigid time limits and long waiting periods before exams.

##### 3.1.3.1 Faster grading and organization

Many students acknowledged that CBT improved efficiency in grading and administration. Musa noted:

*"One major advantage of CBT is that we don’t experience issues like missing scripts, which sometimes happens with paper-based tests."*

Patience also agreed:

*"Yes, because the system marks the exams, making it easier for the lecturer. For me, at the end of the day, I know that if I select the correct answer, I will get my result immediately. There are no missing scripts."*

This aligns with TAM’s perspective that technology is perceived as useful when it simplifies tasks and increases efficiency. Since CBT automates grading and record-keeping, it provides clear advantages for lecturers and administrative staff.

##### 3.1.3.2 Strict time limits and inflexibility

Despite its efficiency, students criticized CBT for its inflexible time limits. Joy pointed out:

*"CBT has a strict time limit. Once the time is up, no extra time is given. However, with paper-based tests, there is some flexibility. Lecturers may grant a little extra time if needed."*

Similarly, Akeem noted:

*"CBT does not give me enough time to carefully evaluate my options, which sometimes makes me second-guess my answers."*

TAM suggests that if users feel constrained by a system, their perception of its usefulness declines. While CBT ensures structured timing, it may cause stress and disadvantage students who need more time to process their responses.

### 3.2 Addressing research question two on the perceived ease of use of CBT among university students

Perceived ease of use refers to students’ perception of how simple, navigable, and user-friendly the CBT platform is, minimizing technical difficulties. We argue that if a system is intuitive and user-friendly, individuals are more likely to adopt and use it. Through open and axial coding, key themes have emerged, reflecting students’ experiences with CBT and their ease (or difficulty) in navigating the system.

#### 3.2.1 Theme 4: The CBT system is generally usable but requires basic computer knowledge

##### 3.2.1.1 Ease of use for computer-literate students

A significant number of students found the CBT system easy to use, describing it as self-explanatory and straightforward. Many noted that having basic computer literacy enhances usability. Aisha emphasized:

*"It is actually self-explanatory and easy to access, as long as you have basic computer knowledge."*

Similarly, Joy confirmed:

*"It is very easy. If you are familiar with computers, it is simple to use."*

This aligns with TAM’s notion that if users find a system intuitive, their likelihood of adoption increases (Davis and Granić, 2024). Since these students already had experience using computers, they did not struggle to navigate the CBT platform.

##### 3.2.1.2 Challenges for students with limited computer knowledge

Some students acknowledged that not everyone is proficient in using computers, which can create difficulties during CBT. Joy suggested that universities should:

*“Give students more practical training on how to use computers. Some students do not know how to operate a computer well, so the school should provide more hands-on training.”*

This suggests that while the system itself may be designed for ease of use, students’ individual competence levels impact their experiences. As per TAM, training and skill development can enhance ease of use perceptions, increasing student confidence and technology adoption.

#### 3.2.2 Theme 5: Persistent technical issues affecting students’ exam experience

##### 3.2.2.1 Frequent server failures and system crashes

While students found CBT usable, technical challenges significantly impacted their experiences. Many reported server failures, login problems, and system crashes that disrupted their exams. Musa recounted a frustrating incident:

*"When I was in my second or third year, there was an incident where we arrived for an exam, but the server failed. We had to wait for over five to six hours before they transferred us to another CBT centre."*

Similarly, Patience described an issue where the system logged her out mid-exam:

*"I was writing an exam, and the system logged me out. I had to wait for the instructor to come and reset everything."*

According to TAM, technical difficulties lower perceived ease of use, making users hesitant to engage with a system. If students encounter repeated disruptions, they may develop a negative perception of CBT, seeing it as unreliable and stressful.

##### 3.2.2.2 Delays and waiting time before exams

Beyond system failures, long waiting times before exams frustrated students. Musa pointed out:

*"When you come to an exam prepared and full of energy, but then have to wait for hours, you get exhausted. By the time the exam finally starts, your concentration and ideas may not be as sharp."*

Long waiting times undermine the efficiency advantage of CBT, contradicting the idea that technology simplifies and speeds up processes. If technical infrastructure is unreliable, students may lose confidence in CBT, reducing its perceived ease of use.

##### 3.2.2.3 Lack of immediate technical support

Students also complained that technical failures were not addressed promptly, leading to further frustration. Musa highlighted:

*"There is no immediate response to technical failures. I think this is one area that needs improvement."*

Similarly, Maryam shared her experience during a JAMB exam:

*"I kept calling the attention of the officials, but they just kept saying, ‘I’m coming.’ I ended up sitting there, doing nothing, and feeling anxious about the possible outcome."*

If users cannot rely on timely technical assistance, they will perceive the system as inefficient and difficult to use, lowering their willingness to accept CBT.

#### 3.2.3 Theme 6: Clear and user-friendly Interface enhances exam completion

##### 3.2.3.1 Simple and well-structured layout

Despite technical challenges, students generally praised the CBT platform’s user interface. They described it as clear, structured, and easy to follow. Joy noted:

*"It is very simple. When you see the interface, you immediately know where to click."*

Similarly, Bosun affirmed:

*"The layout is simple and straightforward, which makes it easy to use."*

According to TAM, a well-designed interface enhances perceived ease of use, making students feel more comfortable with the system.

##### 3.2.3.2 Navigation flexibility improves experience

Students also appreciated navigation flexibility, such as the ability to skip questions and review answers before submission. Musa stated:

*"Yes, you can review and change your answers before submitting the exam."*

Kola described an important feature:

*"There is a section on the right side of the screen that highlights answered and unanswered questions. By clicking on a number, I can quickly navigate to any question without having to scroll through the entire test."*

This feature enhances the perceived usability of CBT, aligning with TAM’s idea that design improvements increase acceptance and satisfaction.

##### 3.2.3.3 Addressing research question three on attitudes of university students toward CBT

In this study, attitudes toward CBT refer to students’ overall perception, preference, comfort, and fairness judgment regarding CBT. Through open and axial coding, we identify recurring themes that explain students’ opinions, experiences, and acceptance of CBT.

#### 3.2.4 Theme 7: Divided preferences between CBT and paper-based testing

One of the strongest themes that emerged from the dataset is that students have mixed feelings about CBT compared to traditional paper-based exams. While some students appreciate the efficiency and organization of CBT, others prefer paper-based testing because it allows them to express their ideas more freely.

##### 3.2.4.1 Students who prefer CBT appreciate its efficiency

Some students find CBT fast, structured, and convenient. For instance, Mark prefers CBT because:

*"My motivation comes from the fact that CBT exams are more organized. All I need to do is go to the CBT centre, take my seat, and start my exam without unnecessary distractions."*

Similarly, Maryam views CBT as technologically advanced and necessary for future learning:

*"The world is advancing technologically, and we should adapt. CBT is more efficient and improves the assessment process."*

This aligns with TAM’s assertion that users are more likely to adopt technology when they perceive it as improving efficiency (Davis and Granić, 2024). The structured nature of CBT, the absence of distractions, and the speed of grading encourage positive attitudes toward its use.

##### 3.2.4.2 Students who prefer paper-based testing value expression and partial credit

On the other hand, some students favor paper-based exams because they allow for open-ended responses and partial credit for effort. Aisha explained her preference:

*"Paper-based testing gives me the opportunity to express my thoughts better. With paper-based tests, I can explain my answers in my own words, whereas with CBT, I can only choose from the given options."*

Similarly, Joy pointed out that:

*"In CBT, if you pick the wrong option, you fail that question. But in a paper-based test, even if your answer is not entirely correct, you may still earn partial marks if it aligns with the question."*

This finding supports TAM’s principle that a technology must meet users’ functional needs to be fully accepted. If students believe that CBT limits their ability to demonstrate knowledge, their attitude toward it becomes negative.

#### 3.2.5 Theme 8: Perceived stress and anxiety in CBT exams

Another crucial factor shaping attitudes toward CBT is the level of stress or anxiety students experience during exams. While some students report feeling comfortable with CBT, others experience technology-induced stress due to technical concerns, the countdown timer, or unfamiliarity with computers.

##### 3.2.5.1 First-time anxiety vs. long-term adaptation

Some students initially felt anxious when taking a CBT exam but became more comfortable with time. Joy described her experience:

*"I only felt tense during my first experience. But after that, like today, I had no tension at all. It was fine."*

This supports TAM’s concept of increasing ease of use with repeated exposure, leading to higher acceptance. As students gain more experience with CBT, their comfort level increases, which improves their attitude toward the system.

##### 3.2.5.2 The countdown timer creates pressure

While CBT eliminates distractions, many students complained about the visible countdown timer, which made them rush through answers. Patience noted:

*"The timer is distracting. It keeps counting down while you are trying to answer questions. It can make you feel rushed and unstable."*

This suggests that CBT’s design elements impact student stress levels, which can influence their overall perception and acceptance of the system. If a feature like the timer creates additional anxiety, students may resist CBT even if it is functionally useful.

##### 3.2.5.3 CBT helps maintain focus due to fewer distractions

Other students reported that CBT helps them focus better because there are fewer distractions compared to traditional paper-based exams. Maryam explained:

*"With paper-based exams, there are more distractions. CBT allows me to concentrate better."*

This aligns with TAM’s assertion that users adopt technology when it simplifies tasks. If students feel that CBT enhances concentration, they are more likely to accept and prefer it over paper-based testing.

#### 3.2.6 Theme 9: Concerns over fairness in CBT

Another major factor influencing students’ attitudes toward CBT is whether they perceive it as a fair method of assessment. Many students raised concerns about fairness due to differences in computer literacy, question randomization, and cheating risks.

##### 3.2.6.1 The digital divide: computer literacy impacts fairness

Some students believe that CBT is unfair to those who lack computer skills. Aisha emphasized:

*"It is fair for students who are comfortable with computers, but not for those who lack computer skills. The university should provide proper training."*

This highlights a major barrier to CBT acceptance: If students feel disadvantaged due to their computer skills, they are less likely to view CBT as fair or beneficial. Bridging the digital divide through training programs can improve attitudes and adoption rates.

##### 3.2.6.2 Randomized questions create perceived inequality

Some students questioned whether CBT provides equal difficulty levels for all students, as the system assigns randomized questions. Aisha noted:

*"The person next to you might get easier questions, while you might get much harder ones."*

This lowers confidence in CBT because students feel disadvantaged if they receive more difficult questions than their peers. TAM suggests that a system must be perceived as fair to be widely accepted, meaning improvements in standardizing difficulty levels could help.

##### 3.2.6.3 CBT prevents traditional cheating but allows impersonation

Many students felt that cheating is more difficult in CBT due to question variation. However, others raised concerns about impersonation. Kola explained:

*"A student can finish their exam, log out, and log in to take the exam for someone else."*

This raises questions about CBT’s security because impersonation is harder to detect compared to traditional exams. If students believe that CBT creates opportunities for certain forms of malpractice, their trust in the system declines.

##### 3.2.6.4 Addressing research question four on the behavioral intention to use CBT

By behavioral intention to use CBT in this study, we mean students’ willingness and likelihood to adopt CBT in future assessments. This study examines students’ willingness to use CBT for future assessments and their suggestions for improvement. Through thematic analysis, we identify key factors influencing their decisions and how these factors align with TAM’s framework.

#### 3.2.7 Theme 10: Willingness to use CBT depends on efficiency and accessibility

One of the strongest themes is that students are willing to continue using CBT if it remains efficient and accessible. Many students appreciate its speed, automation, and structured assessment process, while others have concerns about technical challenges and question format limitations.

##### 3.2.7.1 Students who see CBT as a future option appreciate its efficiency

Several students highlighted that CBT simplifies the examination process and reduces the workload for lecturers. Musa emphasized this point:

*"Yes, CBT is easier in terms of administration and grading. It reduces the workload of lecturers by eliminating manual script marking."*

Similarly, Maryam, a Computer Science student, expressed her confidence in CBT:

*"Definitely, As a Computer Science student, I see myself using CBT more in the future because it is more efficient than traditional exams."*

These statements align with TAM’s principle that perceived usefulness increases technology adoption (Davis and Granić, 2024). Students who recognize the efficiency of CBT in streamlining the grading process and eliminating paperwork are more inclined to continue using it.

##### 3.2.7.2 Concerns about CBT’s reliability affect future use

Some students, however, hesitate to continue using CBT due to technical failures and logistical issues. Mark, for instance, noted:

*"The only factor that would make me avoid CBT in the future is the stress caused by a lack of adequate facilities. Currently, there are not enough computers to accommodate all students, which causes delays and disruptions."*

Maryam also pointed out how technical issues can be discouraging:

*"If technical issues with computers continue, it could discourage me from using CBT. System malfunctions need to be addressed."*

According to TAM, external factors such as system reliability influence technology acceptance. If students continue experiencing technical failures, they may abandon CBT in favor of paper-based exams.

#### 3.2.8 Theme 11: Suggested improvements to enhance CBT adoption

Students provided various suggestions to improve the CBT system, indicating that their willingness to continue using CBT is dependent on whether improvements are made.

##### 3.2.8.1 Expanding CBT centers and increasing computer availability

A common complaint was that limited CBT centers and insufficient computers caused long delays. Aisha emphasized the need for expansion:

*"The university should build more CBT centres to accommodate the increasing number of students."*

Joy described how the lack of computers led to delays:

*"They should provide more computers. For example, today, our exam was supposed to start at 2:30 PM, but we took it around 4:00 PM because there were not enough computers."*

Similarly, Patience called for more facilities to accommodate student demand:

*"More computers and more CBT centres to accommodate the large number of students."*

This aligns with TAM’s principle that external conditions influence technology adoption. If students experience overcrowding and long wait times, their motivation to use CBT decreases.

##### 3.2.8.2 Improving technical performance and IT support

Many students requested better servers and quicker IT responses to reduce system crashes and exam delays. Musa was particularly frustrated with this issue:

*"The main improvement needed is an immediate response to technical issues. The university should also provide more CBT centres to reduce overcrowding."*

Similarly, Patience confirmed that server failures are common:

*"Yes. I have never written an exam where no one had a system issue."*

According to TAM, perceived ease of use is crucial for adoption. If students struggle with technical failures, their overall experience is frustrating, making them less likely to continue using CBT.

##### 3.2.8.3 Enhancing comfort for students during CBT exams

Some students mentioned that standing for long periods before an exam negatively affects their experience. Aisha suggested:

*"The university should provide seating for students waiting outside before the exams. Standing for long periods before an exam can be exhausting."*

Similarly, Patience emphasized:

*"More CBT centres, better servers, and improved ventilation in exam halls."*

These suggestions indicate that the physical exam environment influences students’ attitudes toward CBT. If exam conditions are uncomfortable, students may prefer traditional exams instead.

##### 3.2.8.4 Preference for open-ended questions as a condition for future use

Some students mentioned that they might be more open to using CBT if it included open-ended questions, rather than just multiple-choice questions. Akeem shared:

*"The main factor is flexibility. If CBT allowed for more theoretical responses instead of just multiple-choice questions, I might consider using it."*

Aisha also suggested:

*"Yes, they should include open-ended questions to give students a chance to explain their answers."*

This supports TAM’s notion that technology must meet users’ functional needs. If students believe CBT does not allow them to demonstrate their knowledge effectively, they may hesitate to use it in the future. Table 2 presents the summary of themes and subthemes that emerge from this study.

**Table 2:** Summary of themes and subthemes from the study.

Theme	Subthemes	Core insight
1. Mixed User Experience with CBT	1.1 Technical challenges 1.2 First-time anxiety vs. adaptation 1.3 Limited exposure	Students experience both convenience and frustration; reliability and familiarity strongly shape perceptions.
2. Perceived Limitations in Academic Assessment	2.1 MCQ limits expression 2.2 Guesswork encouraged	CBT’s MCQ format restricts deep expression and may reduce the validity of the assessment.
3. Efficiency vs. Time Constraints	3.1 Faster grading 3.2 Strict timing	CBT is efficient administratively, but rigid timing and delays can undermine performance.
4. Usability Dependent on Computer Literacy	4.1 Easy for literate users 4.2 Difficult for low-literacy users	Basic skills determine ease of use; training greatly influences user confidence.
5. Persistent Technical Issues	5.1 Server failures 5.2 Long waiting times 5.3 Lack of support	Technical breakdowns and slow support reduce trust and ease of use.
6. User-Friendly Interface Enhances Completion	6.1 Clear layout 6.2 Flexible navigation	Simple interface and navigation features increase usability and reduce anxiety.
7. Divided Preferences Between CBT and PBT	(Various preference factors)	Students are split in loyalty to CBT vs. paper tests, based on fairness, familiarity, and test type.

The thematic structure presented in Table 1 synthesizes students’ perceptions of CBT across usefulness, ease of use, attitudes, and behavioral intention. The themes collectively highlight how technical reliability, assessment format, computer literacy, and perceived fairness interact to shape students’ acceptance of CBT. These interconnected perspectives provide a coherent foundation for interpreting students’ digital assessment experiences within the TAM framework. The discussion of these results and their alignments in addressing research questions are presented in the subsequent section.

## 4 Discussion

### 4.1 On the perceived usefulness of CBT among undergraduate students

The findings of this study reveal a mixed perspective on the perceived usefulness of CBT among university students, shaped by both its advantages and limitations. The first major theme highlights the diversity in students’ experiences. While some students found CBT efficient and convenient, others encountered technical challenges, such as poor network connectivity and system malfunctions, which negatively impacted their perception of its usefulness (Perry et al., 2022; Rabiu et al., 2020). In line with previous studies (e.g., Abdulkareem and Lennon, 2023; Malec, 2020), the findings of this study show that first-time users often experienced anxiety, but familiarity over time improved their confidence and ease of use. However, limited exposure to CBT in university settings hindered some students from fully adapting to the system, indicating that consistent implementation could enhance their perceived usefulness. These findings align with the TAM, which suggests that a technology’s perceived ease of use and reliability significantly influence its acceptance (Davis and Granić, 2024).

A deeper analysis of the students’ narratives further reveals that perceived usefulness is strongly shaped by students’ moment-to-moment interaction with the system and institutional conditions surrounding its deployment. Many of the concerns raised, such as unstable servers, slow login processes, and rigid question formats, directly undermined students’ beliefs about whether CBT could genuinely support their academic performance. Thus, usefulness was not conceptualized merely as efficiency or speed, but as the system’s capacity to provide a fair, valid, and supportive assessment environment. In this sense, perceived usefulness emerged as an outcome of students’ accumulated experiences across multiple encounters with CBT, ranging from system reliability to the cognitive demands imposed by multiple-choice-only assessments.

The second theme raises concerns about CBT’s reliance on multiple-choice questions, which students believed restricted their ability to express knowledge comprehensively. Many felt that paper-based exams allowed for partial credit on open-ended questions, whereas CBT’s rigid format encouraged guessing rather than deep understanding (Pengelley et al., 2025). This directly impacts students’ perceptions of whether CBT truly enhances academic performance. The final theme underscores the trade-off between CBT’s speed and automation benefits versus its rigid time limits. Students appreciated faster grading and the elimination of administrative errors like missing scripts, making the system efficient. However, the strict time constraints and lack of flexibility added stress, potentially affecting performance (Weirich et al., 2024). In line with previous studies (e.g., Mcclelland and Cuevas, 2020; Yeboah, 2023), the findings of this study indicate that while CBT enhances efficiency, its perceived usefulness could be improved by addressing concerns related to assessment formats and time flexibility.

### 4.2 On the perceived ease of use of CBT among undergraduate students

The findings of this study suggest that university students generally perceive CBT as user-friendly, but their level of computer literacy significantly influences their perceptions. The first major theme reveals that students who are proficient in using computers find the system intuitive and easy to navigate. They describe it as self-explanatory, reinforcing TAM’s principle that ease of use increases adoption (Davis and Granić, 2024). However, students with limited computer knowledge struggle with CBT, leading to frustration (Nurhikmah and Ervianti, 2021). Some students suggested that universities provide hands-on training to improve familiarity, reinforcing the idea that proper skill development can enhance ease of use and confidence in technology (Zakariya et al., 2024). This indicates that while CBT is not inherently difficult, students’ prior exposure to computers plays a crucial role in shaping their attitudes.

Despite its usability, students reported persistent technical issues that negatively impacted their perceptions of the ease of use of CBT. The second major theme in this section highlights frequent server failures, system crashes, and long waiting times before exams. These challenges frustrate students, making CBT appear unreliable and stressful. Delays and lack of immediate technical support further exacerbate the issue, reducing students’ trust in the system as pointed out in the literature (Emdas and Alruwaili, 2021; Schellekens et al., 2021). According to TAM, when users encounter disruptions, their perception of the system’s ease of use declines (Davis and Granić, 2024). However, the final theme in this section shows that students appreciate CBT’s structured layout and navigation flexibility. Features such as question review options, and a clear interface design make the system more manageable.

### 4.3 On the attitudes of undergraduate students toward CBT

The findings of this study show that university students have mixed attitudes toward computer-based testing (CBT), with preferences influenced by efficiency, fairness, and exam stress. Some students appreciate CBT for its speed, structure, and alignment with technological advancements, making exams more organized and efficient (Pengelley et al., 2025; Syahbrudin et al., 2025). Further, the findings indicate that CBT minimizes administrative errors such as missing scripts and provides instant feedback, which enhances students’ learning experience. Stress levels also shape attitudes, as some students become more comfortable with CBT over time, while others struggle with features like the countdown timer, which increases anxiety. This aligns with findings that first-time users of digital assessments often experience stress, but familiarity improves confidence and ease of use (Syahbrudin et al., 2025). Despite this, many students value CBT’s ability to minimize distractions, helping them maintain focus during exams.

Fairness concerns further influence student attitudes toward CBT. Some students believe CBT disadvantages those with limited computer skills, highlighting the need for training programs to bridge the digital divide (Perry et al., 2022). Additionally, question randomization creates concerns about inconsistent difficulty levels, making some students feel unfairly assessed. While CBT reduces traditional cheating methods, impersonation remains a major concern, undermining confidence in the system’s integrity. Since TAM emphasizes that perceived fairness affects technology adoption, addressing these issues could improve student acceptance and overall attitudes toward CBT (Davis and Granić, 2024).

### 4.4 On the behavioral intention of undergraduate students to use CBT

The findings of this study reveal that university students’ behavioral intention to use CBT depends on its efficiency, accessibility, and system reliability. Many students appreciate CBT for its automation, faster grading, and reduced lecturer workload, aligning with TAM’s principle that perceived usefulness increases adoption (Davis and Granić, 2024). Some students, particularly those in technology-related fields, see CBT as a long-term solution due to its efficiency. However, in line with previous studies (e.g., Azor and Edna, 2019; Perry et al., 2022), concerns about technical failures, inadequate facilities, and limited question formats deter some students from fully embracing CBT. If system malfunctions persist, students may prefer paper-based exams. The reliability of the CBT system plays a crucial role in determining students’ willingness to continue using it.

Students suggested improvements to enhance CBT adoption, including expanding CBT centers, increasing computer availability, and improving IT support to minimize delays and technical issues. Additionally, they emphasized the need for better seating and ventilation in exam halls, as physical discomfort negatively impacts their experience. Some students also preferred open-ended questions rather than multiple-choice formats, suggesting that CBT should allow for more expressive responses. According to TAM, technology must align with users’ functional needs for successful adoption. If these improvements are implemented, students will likely continue using CBT in future assessments.

It is crucial to note that the qualitative narratives from students reveal meaningful interactions among the core constructs that extend the explanatory power of TAM in the context of CBT. The data show that perceived usefulness and perceived ease of use are not independent influences but mutually reinforcing in shaping students’ attitudes. For instance, students who initially struggled with navigating the CBT interface reported diminished confidence in the system’s ability to support fair assessment. Their difficulties with system navigation (ease of use) translated into skepticism about whether CBT could adequately reflect their true academic abilities (usefulness). Conversely, students who described the interface as intuitive and the processes as straightforward were also more likely to express positive beliefs about the efficiency, accuracy, and timeliness of CBT results. This indicates that ease of use operates as a precondition for usefulness, suggesting a hierarchical rather than parallel relationship between the constructs within this educational assessment context.

The findings further reveal that attitudes toward CBT emerge from the interplay between students’ actual interactions with the system and the institutional conditions within which CBT is deployed. Technical failures, inadequate infrastructure, and limited question formats weakened students’ perceptions of both usefulness and ease of use, ultimately shaping negative attitudes and lowering behavioral intentions. Notably, students who experienced smooth, uninterrupted CBT sessions tended to attribute fairness, reliability, and academic support to the system, which demonstrates how positive experiences created a virtuous cycle that strengthened all three TAM constructs. These differences underscore that behavioral intention is not solely a product of individual perception but a reflection of how effectively the institutional environment supports the technology’s intended functions. Thus, TAM in this context operates less as a static classification model and more as a dynamic lens that captures the evolving relationships among technical reliability, student competence, assessment format, and institutional preparedness.

## 5 Conclusion

This study provides a novel understanding of university students’ experiences with CBT in Nigeria while addressing key gaps in the literature by applying the TAM within a qualitative research framework. By investigating perceived usefulness, ease of use, attitudes toward use, and behavioral intention of undergraduate students toward CBT, this research moves beyond the superficial survey-based approaches of prior studies (e.g., Azor and Edna, 2019; Shobayo et al., 2023; Usman and Olaleye, 2022) and offers deeper insights into students’ cognitive and emotional responses to CBT. The findings confirm that students appreciate CBT’s efficiency, automation, and structured format, technical challenges, digital anxiety, and assessment inflexibility hinder their perceived ease of use. This reinforces TAM’s assertion that ease of use directly impacts technology acceptance. Furthermore, students with higher computer literacy demonstrate greater adaptability, whereas those with limited digital skills struggle, emphasizing the role of digital readiness in shaping attitudes toward CBT.

Unlike previous research (e.g., Ukwueze and Uzoagba, 2021) that misapplies general ICT adoption frameworks; this study situates its findings within TAM, demonstrating that technology adoption in education is influenced by both individual factors (e.g., digital literacy) and systemic challenges (e.g., fairness in question randomization). The study’s insights have practical implications for higher education institutions and policymakers. To reduce digital anxiety and improve technological readiness, universities should integrate a mandatory digital literacy module into the first-year introduction to computing courses, which will include hands-on practice tests on the CBT platform. Additionally, assessment policies should encourage instructors to design CBTs that incorporate a mix of multiple-choice, true/false, and short-answer response formats to allow for a more comprehensive evaluation of student knowledge and accommodate different learning styles. These measures will ensure a more inclusive and student-friendly approach to digital assessments.

By addressing methodological and theoretical gaps, this study provides a theory-driven, in-depth exploration of CBT adoption and offers practical recommendations for enhancing digital assessments. Beyond the practical implications, this study makes a significant theoretical contribution by extending the explanatory power of the TAM within the context of digital assessment in Africa. The qualitative evidence demonstrates that traditional TAM constructs, e.g., perceived usefulness and perceived ease of use, do not fully capture the complexities that shape students’ acceptance of CBT. In particular, the findings show that perceptions of fairness, system integrity, assessment authenticity, and infrastructural reliability operate as influential determinants that mediate students’ cognitive and emotional responses to CBT. These factors consistently appeared in students’ narratives, indicating that technology acceptance in high-stakes assessment environments is deeply intertwined with contextual realities such as intermittent technical failures, variation in item difficulty due to randomization, and inequities arising from differential access to digital resources.

Accordingly, the study proposes that fairness perception and system reliability should be considered critical extensions of the TAM when applied to computer-based assessment in resource-constrained environments. These emergent constructs broaden the model’s relevance by demonstrating that students’ intentions are not shaped solely by usability and utility, but also by whether the technological system is perceived as equitable, trustworthy, and capable of supporting valid assessment outcomes. This theoretical refinement positions TAM as a more context-responsive framework for understanding technology adoption in African higher education, where infrastructural limitations and digital inequalities remain persistent challenges. Future adaptations of TAM in educational research should therefore incorporate these additional constructs to provide a more robust explanation of student acceptance of digital assessment systems. While CBT remains a valuable tool for modern education, optimizing its implementation requires bridging digital gaps, refining assessment methodologies, and ensuring equitable access for all students. Future research should explore adaptive testing mechanisms, lecturers’ perspectives, and the long-term impact of CBT on learning outcomes to develop a more efficient and pedagogically effective digital assessment system.

### 5.1 Limitations of the study

Although this study provides meaningful insights into undergraduate students’ perceptions of CBT through the lens of the TAM, several limitations must be acknowledged. First, the study is based on a relatively small sample of nine students drawn from a single university. While phenomenological research does not typically aim for statistical generalization, the limited sample size and institutional homogeneity restrict the breadth of perspectives captured. As a result, the transferability of the findings to other higher education contexts in Nigeria is constrained. Including students from different universities, e.g., federal, state, private, and specialized institutions, could offer a more comprehensive understanding of how contextual differences shape perceptions of CBT. Second, while we acknowledged that purposive sampling could enable the selection of participants with direct CBT experience, the sample lacked broader demographic and cultural diversity. All participants were drawn from a single geographical region, and three of them did not specify their level of study, which may have influenced classification decisions and subsequent subgroup analyses. These limitations suggest that the students’ experiences reported here may not reflect the full range of perceptions held across Nigeria’s diverse higher education landscape. Further, we acknowledge a lack of comparative analysis of students’ perceptions across gender and course of study. This subgroup comparative analysis may provide further insights into the findings of the research. As such, we recommend further investigations in this direction. Lastly, the study relied on short semi-structured interviews lasting 17–22 min. This duration, though sufficient for capturing surface-level experiences, may have restricted the depth of phenomenological inquiry desired for exploring complex constructs such as perceived usefulness, ease of use, trust, and behavioral intention. Future research should consider conducting longer or multiple interview sessions to achieve richer data saturation. These limitations indicate that while the study offers valuable initial insights into students’ experiences with CBT, further research involving larger, more diverse, and multi-institutional samples is necessary to strengthen the transferability and robustness of the findings.

## Data Availability

The raw data supporting the conclusions of this article will be made available by the authors, without undue reservation.
