# Correction: Computer-based testing in higher education: a phenomenology investigation into undergraduate students' perspectives through the technology acceptance model

**DOI:** 10.3389/fpsyg.2026.1810151

**Published:** 2026-02-24

**Authors:** Yusuf Feyisara Zakariya, Sarah Bader Alotaibi, Jawaher Saud Alrashood, Tahani Mohammed Alrosaa

**Affiliations:** 1 Department of Science Education, Ahmadu Bello University, Zaria, Nigeria; 2 Department of Mathematical Sciences, University of Agder, Kristiansand, Norway; 3 Department of Teaching and Learning, College of Education and Human Development, Princess Nourah Bint Abdulrahman University, Riyadh, Saudi Arabia

**Keywords:** attitudes toward computer-based testing, behavioral intention, CBT anxiety, ChatGPT, students' performance, technology acceptance model

The funder Princess Nourah bint Abdulrahman University Researchers Supporting, Project number (PNURSP2025R583) was erroneously omitted. The correct funding statement reads:

The author(s) declared that financial support was received for this work and/or its publication. The University of Agder covered the APC for the publication of this article. The research was funded by Princess Nourah bint Abdulrahman University Researchers Supporting Project number (PNURSP2025R583), Princess Nourah bint Abdulrahman University, Riyadh, Saudi Arabia.

The original version of this article has been updated.

