# Supplementary material for: Computer-based testing in higher education: a phenomenology investigation into undergraduate students’ perspectives through the technology acceptance model
Source: Front Psychol. 2026 Jan 22;17:1602964. doi: 10.3389/fpsyg.2026.1602964 (PMC12874087; doi:10.3389/fpsyg.2026.1602964)
Supplement: Supplementary file 1 [file Data_Sheet_1.docx]

**Appendix A: AI-Assisted Qualitative Data Analysis Framework**

To ensure transparency and replicability, this appendix provides the key prompts and iterative procedures used in the AI-assisted qualitative data analysis.

**Prompts Used for Open and Axial Coding (Example: Research Question 1 – Perceived Usefulness)**

**Prompt 1: Initial Open Coding**

*“Here is a full transcript for a qualitative research dataset aimed at investigating students’ acceptance of computer-based testing in a higher institution. Using the first dimension of the Technology Acceptance Model (perceived usefulness of CBT: general experience with CBT, effectiveness of CBT, and time and efficiency) as an analytical framework, conduct open coding of the dataset. Identify meaningful units, generate open codes, and retain direct participant quotations. Make your coding process explicit.”*

**Iterative Refinement:**

1. When vague or overly general codes were generated, the prompt was amended to request more fine-grained codes and clearer differentiation.
2. Additional clarifications were added, such as:

*“Avoid summarising; extract specific codes tied to direct quotes.”*
*“Do not cluster codes at this stage—generate them as discrete units.”*

**Prompt 2: Axial Coding**

*“Using the refined open codes previously generated and the Technology Acceptance Model as a guiding framework, cluster related codes into categories and develop overarching themes that address the research question. Ensure that categories are conceptually distinct and grounded in the participants’ direct quotations.”*

**Iterative Refinement:**

1. When overlapping categories appeared, a second instruction was issued:

*“Reassign ambiguous codes to the most conceptually appropriate category and justify the decision.”*

1. When themes appeared too broad:

*“Narrow themes to ensure each reflects a coherent set of related meanings.”*

**Human Cross-Validation Procedures**

1. **Researcher Review of AI Output**
   1. Each set of AI-generated codes and themes was reviewed line-by-line by two human coders.
   2. Misinterpretations, missing nuances, or codes unrelated to the original data were flagged.
2. **Resolution of Discrepancies:** Human coders discussed and resolved inconsistencies, making manual adjustments where necessary.
3. **Final Verification Against Raw Data:** Themes were checked again against the verbatim transcripts to confirm grounding in the dataset.
4. **Documentation:** All coding decisions, refinements, and changes to the prompts were documented for auditability.

**Steps Taken to Reduce Bias and Inaccuracy**

1. Use of multiple iterations to refine AI outputs.
2. Human oversight at every stage of coding.
3. Independent cross-checking by two researchers.
4. Anchoring all coding decisions in the TAM framework and raw transcripts.
5. Avoidance of accepting AI-generated categories without human verification.
